# Disparity in socio-economic status explains the pattern of self-medication of antibiotics in India: understanding from game-theoretic perspective

**DOI:** 10.1098/rsos.211872

**Published:** 2022-02-09

**Authors:** Bhawna Malik, Habib Hasan Farooqui, Samit Bhattacharyya

**Affiliations:** ^1^ Disease Modelling Lab, Mathematics, School of Natural Sciences, Shiv Nadar University, Greater Noida, India; ^2^ Indian Institute of Public Health, Public Health Foundation of India, Delhi, India; ^3^ College of Medicine, Qatar University, Doha, Qatar

**Keywords:** antimicrobial resistance, self-medication, socio-economic growth, human behaviour, evolutionary game theory

## Abstract

The emergence of antimicrobial resistance has raised great concern for public health in many lower-income countries including India. Socio-economic determinants like poverty, health expenditure and awareness accelerate this emergence by influencing individuals' attitudes and healthcare practices such as *self-medication*. This self-medication practice is highly prevalent in many countries, where antibiotics are available without prescriptions. Thus, complex dynamics of drug- resistance driven by economy, human behaviour, and disease epidemiology poses a serious threat to the community, which has been less emphasized in prior studies. Here, we formulate a game-theoretic model of human choices in self-medication integrating economic growth and disease transmission processes. We show that this adaptive behaviour emerges spontaneously in the population through a self-reinforcing process and continual feedback from the economy, resulting in the emergence of resistance as externalities of human choice under resource constraints situations. We identify that the disparity between social-optimum and individual interest in self-medication is primarily driven by the effectiveness of treatment, health awareness and public health interventions. Frequent multiple-peaks of resistant strains are also observed when individuals imitate others more readily and self-medication is more likely. Our model exemplifies that timely public health intervention for financial risk protection, and antibiotic stewardship policies can improve the epidemiological situation and prevent economic collapse.

## 1. Introduction

*Self-medication* (SM) is a global phenomenon and a potential contributor to antimicrobial resistance worldwide, especially in LICs and LMICs [1–4]. It is a common behavioural practice that includes self-diagnosis of illness and the utilization of antibiotics to treat without seeking proper medical suggestions [5]. Individuals adopt and practice self-medication to avoid expensive and lengthy treatment procedures. Poor socio-economic status, lengthy diagnostic processes, expensive medicines, lack of education and awareness are key factors contributing to such behavioural practice [6]. In economically destitute communities, unavailability and inaccessibility of healthcare facilities also motivate such adaptive social behaviour [1,7]. It was found that 50% of purchased antibiotics in South Asian countries like India, Nepal, Bangladesh and Pakistan is through Over-the-Counter (OTC) drug sales, which plays a crucial role in fostering self-medication [3,8–10]. Irrational use of antibiotics without medical supervision may result in improper diagnosis and treatment, resulting in greater probability of delay in proper health care, leading to microbial resistance and increased morbidity [11–13].

**Figure 1.**
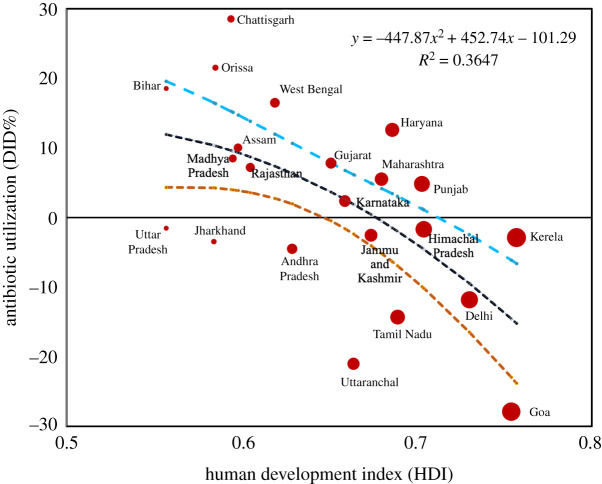
Empirical association between community-level utilization of antibiotics (2012–2016) [21] and the Human Development Index (HDI) (2015) of different states of India. Antibiotic use and self-medication are higher in states with limited access to healthcare facilities, low *per capita* income, and a lack of awareness and literacy. A polynomial regression shown in black dotted line indicates a negative relationship between these two variables. The other two dotted lines in orange and cyan exhibit 95% confidence interval. The HDI data are available from Global Data Lab: https://globaldatalab.org/shdi/shdi/ (accessed on 25 July 2021). See electronic supplementary material for more details.

In resource-limited countries, there is also a high prevalence of antimicrobial self-medication [14,15]. For example, one in three households in Bangladesh, Vietnam, Thailand, Ghana, Mozambique and South Africa reported obtaining antibiotics without a prescription [16]. In previous and recent literature, it was also reported that approximately 80% of all antibiotics are bought without prescription in developing nations [14,17–20]. The reciprocal relationship between OTC antibiotic use and socio-economic development is also visible in Indian states (figure 1). States with inadequate access to healthcare facilities, low *per capita* income, lack of awareness and literacy show higher antibiotic utilization and self-medication (electronic supplementary material, figure S1). Countries like Brazil, Russia, India, China and South Africa (BRICS countries) are developing countries that showed their highest drug consumption from 2000 to 2010 with India as the first and China in second position. It was estimated that approximately 16.8% of the total medicine sold in India between 2013 and 2014 was antibiotics, worth approximately $12.6 billion [21].

Community-level antimicrobial self-medication has a direct correlation with the emergence and burden of resistance. Our recent survey study shows resistance of *Escherichia coli* and *Klebsiella pneumoniae* in India has a strong positive correlation with the steady increase of *Carbapenems* utilization from 2007 to 2018 (details are given in electronic supplementary material, text and figure S2). This widespread inappropriate use of antibiotics, on the other hand, comes at a high cost to a country’s economy, particularly in the case of LICs or LMICs [22–25]. For example, it was estimated that resistance resulted in as much as $20 billion indirect costs in high-income nations, with $35 billion societal costs for lost productivity each year in the USA alone [26]. As predicted, it would lose $1.1–3.8 of its annual gross domestic product (GDP) due to resistance by 2050 [27]. A World Bank estimate indicates that LICs and LMICs will suffer more in comparison to developed nations, stating that a total of 24 million people will be forced into extreme poverty due to resistance by 2030 [27,28].

Compartmental models have been used to study epidemiological aspects of the emergence of drug-resistance [6,29–35]. There are also game-theoretic models to describe human behavioural interactions in infectious disease modelling, especially in vaccination choice [36–38]. However, less emphasis has been placed so far on modelling human behaviour in the emergence of drug-resistance. To mention a few, Kamal Jnawali *et al.* [39] have developed a classical two-player stochastic game theoretical model showing that strategic interactions could strongly influence a population’s choice of antiviral drug use policy. Fu & Chen [40] have shown how social learning may help prescribing behaviour of physicians to promote the social optimum of antibiotic consumption. Coleman *et al*. [41] have also studied this social dilemma among doctors in prescribing antibiotic drugs, and highlighted that rational doctors are always motivated to attain the best outcomes for their own patients, irrespective of the impact on the community, leading to situations like the tragedy of the commons. Apart from these, a paper by Conlin *et al*. [42] used evolutionary game theory to illustrate that the population dynamics found in microbial experiments are predicted by different two-strategy, two-player games. Von Neumann *et al.* [43] studied a coordination game between a leader population and a follower population to show how imitation can lead to an economy in a poverty trap. However, the emergence of antimicrobial resistance is an ecological phenomenon—the result of a complex interplay among disease prevalence, socio-economic conditions and antibiotic utilization through self-medication [44–46]. To avoid high treatment costs, lengthy diagnosis and expensive medicines, irrational and inappropriate use of antibiotics driven by individual self-interest often crosses the social-optimum of antibiotic consumption, accelerating the emergence of drug-resistance in the population [47–49]. Thus, self-medication can be seen as a tragedy of the commons, and therefore, it is of significant public health interest to understand and manage antibiotic resistance from this behavioural perspective. To date, there is no modelling study that highlights human behaviour in self-medication and its impact on community-acquired drug-resistance.

In this paper, we use evolutionary game theory to model the co-evolving dynamics of human behaviour in self-medication and the emergence of resistance. Three components—ecology of infectious disease, socio-economic growth and antibiotic misuse—generate a self-reinforcing cycle under continuous feedback on each other (electronic supplementary material, figure S3). In our previous work [6], we explored the feedback system assuming a hypothetical relationship between economic growth and antibiotic misuse. Here, in this research work, we investigate the emergence of resistance under the mechanistic frame of human behaviour (i.e. *self-medication*) that mediates antibiotic misuse. To the best of our knowledge, this is the first study that examines individual self-medication behaviour from various epidemiological and socio-economic perspectives, as well as its implications for the emergence of antibiotic resistance. Exploring the dynamics of resistance through the lens of human behaviour allows us to better understand the relationship between population-level medication behaviour and its consequences on the emergence of drug-resistance, which provides insight into developing context-specific interventions to address the population-level drug-resistance problem. For example, we compute social-optimum self-medication and determine its proportional disparity with individual interest depending on key parameters like treatment effectiveness, health awareness and socio-economic conditions. We also find that social learning in self-medication has a significant influence on the pattern of emergence. Our analyses point out that timely public health initiatives can break this self-reinforcing cycle, and recover the population from economic downfall due to antibiotic drug-resistance—a result of the public health importance in controlling antibiotic drug-resistance.

## 2. Model framework

Here, we formulate the individual choice of self-medication as an evolutionary game in the population. Once infected with a pathogen, individuals develop symptoms of variable order. For example, individuals infected with a sensitive strain report mild symptoms, whereas severe symptoms develop upon infection by a resistant strain. The players of the game are individuals who develop mild symptoms. They decide after diagnosis whether to adopt self-medication or to choose a proper medical consultation and follow-up, referred to as hospital treatment. Individuals' perception of either opting for self-medication or choosing proper hospital treatment evolves with time depending on prevalence of resistant strain in the population. We model this decision-making as a two-strategy pairwise contest: we define the population state as **x(t)** = (*f*_*s*_, *f*_*T*_), and *S* ={SM,HT} denotes strategy set comprising self-medication (SM) and hospital treatment (HT), where *f*_*s*_(*t*) is the fraction of population using strategy SM (self-medication) per unit time (day) and *f*_*T*_(*t*) = 1 − *f*_*s*_ be the fraction of population using strategy HT (hospital treatment) per unit time (day). The perceived payoff for adopting a self-medication strategy is

2.1 $$\pi (SM,x)=-r_{\mathrm{sev}}mz,$$

where *r*_sev_ is the perceived penalty due to severity of infection by resistant strain, and parameter *m* (∈ (0, 1)) is the fraction quantifying the sensitivity of adopting hospital treatment to change in resistant prevalence. The negative sign indicates the cost incurred upon infection with resistant strain. The perceived payoff for adopting hospital treatment is given by

2.2 $$\pi (\mathrm{HT},x)=g(r_{H},h,h_{o}),$$

where *g*(.) is a function of economic growth *h*, education and awareness *h*_*o*_, and treatment cost *r*_*H*_ that includes cost of hospital visits, diagnostic testing, medicines, loss of wages, etc. Here, we assume that individuals use a ‘rule of thumb’ to estimate the treatment cost and also probability that they become infected, instead of having a perfect knowledge of it. When △E=π(SM,x)−π(HT,x)>0 implies individuals switch to self-medication, whereas △E<0 indicates switching to hospital treatment. If an individual samples others at a rate *σ*, and switches to a strategy with a proportionality constant *ρ*, the growth equation of the population opting for self-medication is

2.3 $$\frac{df_{s}}{dt}=(1-f_{s})\cdot \sigma f_{s}\cdot \rho △E=\kappa f_{s}(1-f_{s})(\pi (\mathrm{SM},x)-\pi (\mathrm{HT},x)),$$

where *κ* = *σρ* is the combined imitation rate. We should note that the same equation (2.3) represents the growth equation of population adopting hospital treatment *f*_*T*_ = 1 − *f*_*s*_.

### 2.1. Integrated model of antibiotic resistance and self-medication

We integrate population ecology of infectious diseases, and self-medication game to develop the model of resistant transmission under antibiotic overuse. We consider a general SIS framework for the disease process. Electronic supplementary material, figure S4 shows the schematic of the model, and table 1 provides a description of the variables and parameters used in the model. Infected individuals reporting mild symptoms can move to either *y*_*T*_ or *y*_*s*_ depending on the choice of a strategy for hospital treatment or self-medication. A detailed description of the model is given in the electronic supplementary material. Including equation (2.3), the behaviour-prevalence model of drug-resistance and self-medication:

2.4 $$\begin{array}{rl} & \frac{dS}{dt}=\mu -\beta S(y+y_{s}+\epsilon y_{T})-\beta^{\prime }Sz+\gamma_{1}y+\gamma_{2}z+\eta_{1}y_{T}+\eta_{2}y_{s}-\mu S,\\ & \frac{dy}{dt}=\beta S(y+y_{s}+\epsilon y_{T})-\theta y-\gamma_{1}y-\mu y,\\ & \frac{dz}{dt}=\beta^{\prime }Sz+\xi (\nu y_{T}+y_{s})z+\sigma (\nu y_{T}+y_{s})-\gamma_{2}z-dz-\mu z,\\ & \frac{dy_{T}}{dt}=\theta (1-f_{s})y-\nu \xi y_{T}z-\nu \sigma y_{T}-\eta_{1}y_{T},\\ & \frac{dy_{s}}{dt}=\theta f_{s}y-\xi y_{s}z-\sigma y_{s}-\eta_{2}y_{s}\\ & \mathrm{and}\;\;\;\;\frac{df_{s}}{dt}=kf_{s}(1-f_{s})△E. \end{array}\}$$

**Table 1.** Baseline parameter values for simulation.

parameters	description	values/range (day^−1^)	reference
*r*_*sev*_	risk from self-medication	5.5	calibrated
*r*_*H*_	risk from hospital treatment	2.5	calibrated
$r_{h}^{c}$	intrinsic growth rate	0.035	[6,50]
$r_{l}^{c}$	*per capita* amount spend on training or education of labour	—	[6,50]
*m*	sensitivity of individual decision	0.7	[40]
*κ*	combined imitation rate	(0.05–0.5)	[37]
*ω*	relative risk	(0.01–1)	
*δ*_1_	rate of capital depreciation		[50]
*β*	transmission rate of sensitive strain	(0.18–0.2)	[40]
*β*′	transmission rate of drug-resistant strain	(0.02–0.08)	[40]
$R_{oy}$	reproduction number for sensitive strains	—	
$R_{oz}$	reproduction number for drug-resistant strain	—	
*γ*_1_	recovery rate of sensitive strains	0.0833	[6,50]
*γ*_2_	recovery rate of drug-resistant strains	0.02	
*μ*	mortality rate	$\frac{1}{\left(55*365\right)}$	[6,50]
*d*	death rate due to resistant	0.00001	
*σ*	mutation rate from sensitive strain to resistant strain	10^−3^	[40]
*ξ*	plasmid transfer rate	(0.05–0.1)	
*ϵ*	reduced probability of infection from treated individuals	(0.2–0.8)	[51]
*θ*	diagnostic rate	(0.02–0.5)	[51]
*ν*	reduced probability of mutation and plasmid transfer for treated individuals	(0.005–0.5)	calibrated
*η*_1_	rapid recovery rate of individuals infected with sensitive strain due to treatment	$\left(\frac{1}{5}-\frac{1}{10}\right)$	[40]
*η*_2_	rapid recovery rate of individuals infected with sensitive strain and taking self-medication	$\left(\frac{1}{20}-\frac{1}{25}\right)$	[40]
*α*	the proportional effect on production and labour of the economic growth from treated individuals	(0–1)	calibrated
*h*_*o*_	education and awareness level	0.5	[6]
*c*_*z*_	*per capita* cost of treatment of resistant strain		calibrated

### 2.2. Economic growth and feedback

We further integrate socio-economic impact with the model 2.4, a similar approach of the integrated system developed by some of the authors [6]. In this integrated system, we also assume that infection has consequences for the economic growth of the population. We use a linear form of Solow’s model to describe economic growth and its interaction with infections:

2.5 $$\frac{dh}{dt}=(r_{h}h+r_{l})-\delta_{1}h-c_{z}z,$$

where *c*_*z*_ is the *per capita* cost to treat individuals infected with resistant strain, and *δ*_1_ is the *per capita* rate of capital depreciation. *r*_*h*_ and *r*_*l*_ are elasticity parameters defining the growth of economy from capital and labour. We assume these two parameters are functions of strain prevalence because infections impede economic production [52–54]:

2.6 $$r_{h}(y,y_{s},y_{T},z)=r_{h}^{c}(1-y)(1-y_{s})(1-\alpha y_{T})(1-z)$$

and

2.7 $$r_{l}(y,y_{s},y_{T},z)=r_{l}^{c}(1-y)(1-y_{s})(1-\alpha y_{T})(1-z).$$

Here, we consider the proportional impact of different disease classes on production and labour related to the economic growth: less impact by treated individuals (denoted by *α* < 1) compared to the other infected populations (*y*(*t*), *y*_*s*_(*t*), *z*(*t*)). Detailed descriptions of models and parameters are given in the electronic supplementary material.

## 3. Results and discussion

### 3.1. Model equilibria and socially optimum self-medication

**Figure 2.**
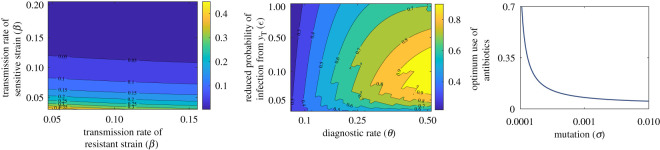
Socially optimal self-medication in different epidemiological parametric regime. The socially optimum values increase with an increase in *β*, *β*′, but decrease with *ϵ*, and *σ*. Parameters values for these plots are given in table 1, except *ξ* = 0, *d* = 0, *ν* = 0.08, *η*_1_ = 1/4. Details are in text.

There are five equilibria of the model including pure self-medication and pure hospital treatment. We use next-generation matrix to compute the basic reproduction ratio and *social-optimum* self-medication (electronic supplementary material, equation S4). Socially optimum self-medication is the limit of community-level antibiotic utilization beyond which the resistant strain emerges and becomes endemic in the population. However, the socially optimum values decrease with an increase in transmission rate (*β*, *β*′), effectiveness of treatment (*ϵ*), and mutation rate (*σ*) (figure 2). Detailed calculations of equilibria, social optimum, and impact of social learning in self-medication are given in the electronic supplementary material.

**Figure 3.**
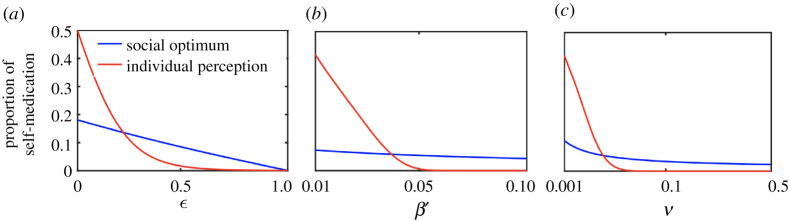
Comparison of social-optimal and individual interest in self-medication against different epidemiological parametric. The figure shows that individual self-medication is substantially greater than social-optimum at lower values of these parameters *β*′, *ϵ* and *ν*. Base parameters values for these plots are given in table 1, except *ω* = 42, *ν* = 0.05, *k* = 0.4, *ξ* = 0, *d* = 0. Details are in text.

To investigate how the disparity between the socially optimum and individual interest in antibiotic utilization changes with critical epidemiological parameter regime, we plot both variables against treatment effectiveness (*ϵ*), relative probability of mutation and plasmid transfer (*ν*) and transmission rate (*β*). At lower values of these parameters, individual self-medication is much higher than social-optimum (figure 3). However, the prevalence drops steeply as the parameter values increase and it even falls behind the social-optimum threshold. These simulations indicate that public health can work towards informing individuals about realistic values of those parameters to improve the control of drug-resistance.

### 3.2. Dynamical regime of individual self-medication

#### 3.2.1. Effectiveness of hospital treatment

Treatment effectiveness influences individual decisions in self-medication by changing emergence and transmission of resistant strains in populations. We have two proxy parameters to measure this: *ϵ*, the intensity of transmission by treated (inappropriately) individuals, and *ν*, the probability of mutation and plasmid transfer from treated (inappropriately) individuals. We investigate this considering the simpler form of *g*, say *g*(*r*_*H*_, *h*, *h*_*o*_) = −*r*_*H*_.

**Figure 4.**
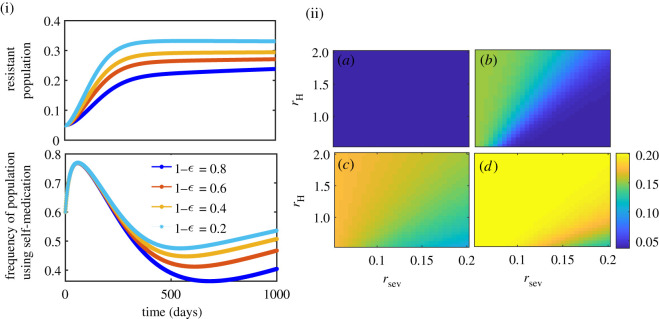
(i) Time series showing the impact of treatment effectiveness (1 − *ϵ*) on resistant population and self-medication behaviour when *r*_*H*_ = 2.5, *r*_sev_ = 5.5, and (ii) endemic prevalence of resistance under different effectiveness: (*a*) 0.8, (*b*) 0.6, (*c*) 0.4 and (*d*) 0.2. Figure exhibits higher effectiveness leading to lower self-medication in the long run, that further leads to lower endemic prevalence of resistant strains. Details are in text.

As observed from figure 4, higher efficacy (1 − *ϵ*) shows lower frequency of self-medication (figure 4(i)), and lower endemic prevalence of resistant population (figure 4(ii)). Higher *ϵ* (i.e. lower effectiveness 1 − *ϵ*) implies higher probability of transmission of sensitive strain due to inappropriate treatment, which in turn increases the frequency of self-medication in the beginning, and hence increases the chance of resistant mutation and transmission. This has been observed in figure 4(i). However, as individuals’ risk perception from infection increases linearly with the resistant strain frequency, the perceived risk increases, and hence individuals switch to treatment strategy later and that suppresses the usage of self-medication and lowers the emergence of resistance. The same is also reflected in the parameter plot *r*_sev_ − *r*_*H*_– endemic prevalence of resistant strain decreases as relative perceived risk *r*_sev_/*r*_*H*_ increases (figure 4(ii)).

A similar dynamics is observed in variation of *ν*, the relative probability of mutation and plasmid transfer from treated individuals. We explored the effect of *ν* under two different recovery periods (indicated by *η*_1_) of treated individuals. At very low relative probability *ν*, frequencies of individuals chose self-medication under different *η*_1_ showing a significant difference, which increases with time. By contrast, the difference in self-medication proportions are negligible when *ν* is higher. The same is observed with a resistant population in the parameter plan of *r*_sev_ − *r*_*H*_. Details have been discussed in electronic supplementary material. These have, however, a very interesting consequence from the public health point of view. Public health authorities should inform people about actual and long-term risks from the drug-resistance. The higher relative risk of infection from resistance can be scaled up with the current effectiveness of the treatment to reduce the emergence and burden of resistance in the population.

#### 3.2.2. Risk perception as a function of income and awareness

The perceived cost of hospital treatment varies with income and awareness in the population [55–57]. For example, individuals with higher income and high awareness about drug-resistance may find the hospital treatment is necessary and perceive the cost lower, whereas a low-income individual with little awareness may consider the same cost higher and hence, ignore the hospital treatment upon infection. This, however, introduces a reciprocal relation of perceived hospital cost with individual income and awareness, impacting further on decision-making in self-medication. To explore this, we improve the payoff function (2.2) by considering *g*(*r*_*H*_, *h*, *h*_*o*_) = *r*_*H*_/(*h*_*o*_ + *h*).

**Figure 5.**
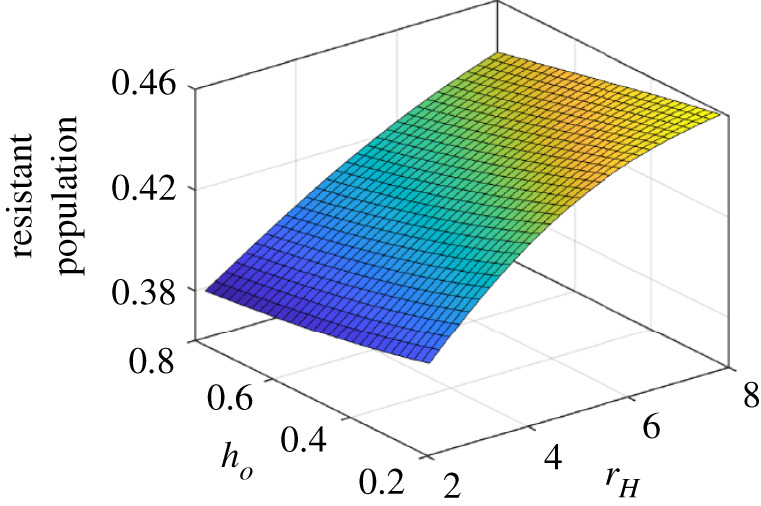
Impact of education and awareness on the emergence of resistance strain in the population. Low value of (*h*_*o*_) signifies higher education and awareness, while high values means lower education and less awareness. The low education drives more self-medication and vice-versa. Along with the baseline values, other parameter values used for this simulation are *r*_sev_ = 2.5, *κ* = 0.0002, *ξ* = 0.05, *σ* = 10^−2^, *θ* = 0.45, *a* = 0.2. For a detailed explanation, see the text.

With an improved version of the payoff function, we plot the resistant population as a function of awareness and hospital cost *r*_*H*_. Figure 5 exhibits that the density of resistant population decreases as *h*_*o*_ increases and *r*_*H*_ decreases. Higher *h*_*o*_ and low *r*_*H*_ decrease frequency of self-medication (see equation (2.2)), thereby reduces the emergence of drug-resistance. As an implication towards public health management of drug-resistance, it indicates that an increase in education and awareness of potential risks from self-medication practices, especially antimicrobial, might help to improve the situation. At the same time, reduced hospital treatment costs might lower the possibility of self-medication and individuals will be inclined to proper treatment upon infection by any strain.

#### 3.2.3. Expected utility as function of success rate

It is very rare to have any empirical data to support how individuals perceive situations and how perceptions evolve over time. The probability of success of strategies and using it in the calculation of expected utility sometimes plays a major role in decision-making, especially in health-related events and health-seeking behaviour. We characterize this scenario by reformulating the payoff function *g* and scaling the perceived hospital treatment cost using treatment effectiveness (1 − *ϵ*). We consider two cases: (a) perceived cost does not depend on the effectiveness of treatment, i.e. *g*(*r*_*H*_, *h*, *h*_*o*_) = *r*_*H*_/(*h*_*o*_ + *h*), and (b) the utility of strategy equals the probability of success × cost of the strategy, i.e. *g*(*r*_*H*_, *h*, *h*_*o*_) = (1 − *ϵ*)*r*_*H*_/(*h*_*o*_ + *h*).

**Figure 6.**
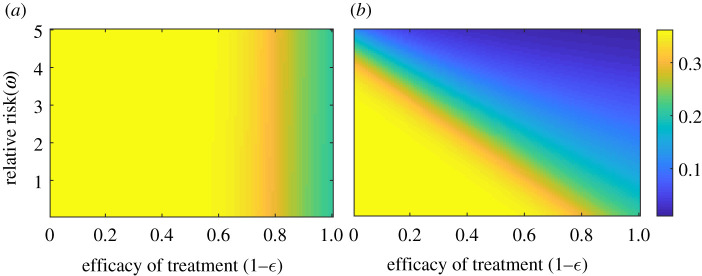
Resistance population density in plane (1 − *ϵ*) − *ω* for two different perceived risk functions with *ω* = *r*_sev_/*r*_*H*_ when: (*a*) the perceived cost is independent of the effectiveness of treatment (*g* = (− *r*_*H*_)/(*h*_*o*_ + *h*)), and (*b*) the utility of strategy equals the probability of success × cost of the strategy (*g* = (− (1 − *ϵ*)*r*_*H*_)/(*h*_*o*_ + *h*)). Emergence of drug-resistance is much lower when efficacy of treatment is considered in the perceived cost of treatment. Along with the baseline values, other parameter values used for this simulation are *r*_*H*_ = 4.5, *r*_sev_ = 2.5, *κ* = 0.01, *ξ* = 0.1, *σ* = 10^−2^. For details, see the text.

When success rate is not considered in calculating the expected utility, the relative risk does not interact directly with treatment efficacy in the emergence of resistant strain in the population (figure 6*a*)). The density of resistant strain does not change with increases in relative risk *ω* ( = *r*_sev_/*r*_*H*_), although it has a little decrease when the effectiveness of the treatment is very high. By contrast, there is a stark difference in the resistance prevalence pattern when effectiveness is considered in the payoff function (equation (2.3)). It multiplies the impact of relative risk in individuals’ decision-making. At the higher effective treatment, a little increase in *r*_sev_ motivates individuals to switch to hospital treatment that reduces the endemic prevalence of resistant (figure 6*b*). Considering the efficacy of treatment or success rate while calculating the expected utility thus reduces the frequency of self-medication. Health authorities should inform the public about the effectiveness of the treatment through media coverage and personal communication so that individuals consider this factor as an important component while estimating their payoff in medical decision-making.

### 3.3. Public health intervention

International organizations (i.e. third party funding) allocate funds in developing countries to fight against drug-resistance. All such allocated budgets may act as an incentive at the individual level to choose proper hospital treatment, and reduces the emergence of resistance in the population by lowering the frequency of self-medication. We explore the case even with a more realistic situation where the budget is released proportional to the current prevalence of resistance—meaning the higher the prevalence, the more proactive the public health authorities are—in allocating funds to initiate a rapid response to the situation. Coupling the incentive of budget allocation in the payoff equation, we have

3.1 $$△E_{1}=\left(-r_{\mathrm{sev}}mz+\frac{r_{H}}{h_{o}+h}-\frac{\zeta z}{a+z}\right),$$

where *ζ* is the maximum budget available per unit time, and *a* is the sensitivity parameter reflecting how likely it is that those third parties are responsive in allocating funds. Lower values of *a* means that parties are more sensitive to the disease prevalence and release more funds.

#### 3.3.1. Impact of intervention

**Figure 7.**
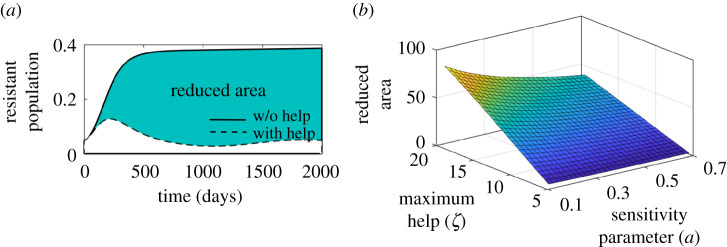
Scenario analysis illustrating sensitivity of public health initiative to reduce the effect of drug-resistance. (*a*) The bold curve depicts the dynamics without intervention, while the dotted curve shows the results after implementing the help. *ζ* = 10 and *a* = 0.5. (*b*) Plot of reduced area (green) bounded by the bold and dotted curve in the given figure for varying *ζ* and *a*. Realizing a high amount of aid and rapid initiative might help better in reducing the prevalence of resistance. Along with the baseline values, other parameter values used for this simulation are *r*_*H*_ = 5.5, *r*_sev_ = 2.5, *κ* = 0.002, $r_{h}^{c}=0.025$. For details, see the text.

Figure 7*a* exhibits time series of dynamics of resistance accumulation with and without public health intervention. It clearly shows that the population colonized with resistant strain is much less when public health prioritizes allocating funds quickly enough during the development of resistance in the population. However, the actual reduction in the burden of resistance depends on when and how much aid is allocated. To quantify this, we plot the area bounded by these two curves (before and after aid) for a range of values *ζ* and *a*. As observed in figure 7*b*, the situation is much improved if the funding is supplied on time, especially on the early level of development of drug-resistance in the community. The decline in the resistant population is moderate with the low value of *a* in spite of the high budget. It is evident that the impact of *a* is higher when the maximum fund *ζ* is higher. Thus, the authority should be providing maximum financial resources as early as possible. Once public health authorities start investing in healthcare services and financial risk protection schemes, the utility gain in switching to HT increases compared to self-medication. This clearly underscores that agencies and policymakers need to be more proactive to combat the situation of antibiotic drug-resistance.

#### 3.3.2. Recovery from economic downfall through public health investments

**Figure 8.**
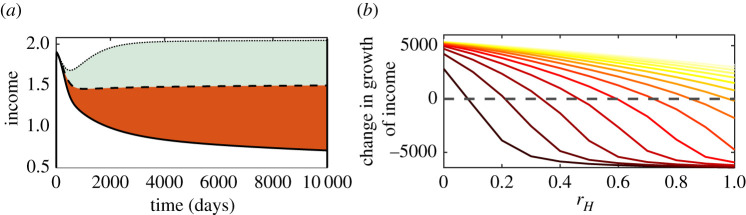
Illustration of public health initiative to prevent population from economic downfall. (*a*) Time series exhibits change in income (*h*) due to different treatment cost *r*_*H*_. The middle curve (*r*_*H*_ = 0.5) is assumed to be the stable and normal state of the income. The upper curve (*r*_*H*_ = 0) shows income accumulation and lower curve (*r*_*H*_ = 1) denotes income depreciation. Area (grey) bounded by upper and middle one is assumed to be positive growth of economy, whereas area (in red) bounded by lower and middle curve is defined as depreciation or loss. (*b*) Curves represent the change in income due to different values of *ζ* = 0 − 15: extreme left curve with dark red colour represents *ζ* = 0 (no aid), whereas extreme right with yellow colour is drawn for *ζ* = 15 (high amount aid). This shows that the growth of income rapidly falls (at *r*_*H*_ = 0.1) below the dotted line when there is no public health aid (i.e. *ζ* = 0). However, with an increase in financial aid, the value of *r*_*H*_ beyond the income becomes negative and shifts towards right. With a sufficient aid (*ζ* = 15), the graph of income never falls below zero, and thus the economy can recover from downfall. Along with the baseline values in table 1, other parameter values used for this simulation are *r*_sev_ = 0.5, *κ* = 0.005, $r_{h}^{c}=0.045$, *h*_*o*_ = 0.6, *a* = 0.8.

Public health funds from a government not only help to reduce the burden of resistance, but also enhance the economic growth of the community and help it recover from financial collapse. The correlation between health and income is allied through a vicious cycle: income impacts health and health affects the income, and in turn, the economy of the population. Many investigations have pointed out that healthcare costs impose a huge economic burden in low and middle-income countries [51,58–61]. The continual feedback between income and health thus eventually leads the population to a poverty trap [52,62]. An external perturbation is necessary to break the self-reinforcing cycle. We investigate this by comparing the change in income (*h*) by varying hospital cost (*r*_*H*_) (figure 8*a*). Low cost (*r*_*H*_ = 0) increases the income, while high cost (*r*_*H*_ = 1) reduces the income from the stable economic growth (at *r*_*H*_ = 0.5). We plot the area bounded by different *r*_*H*_ curves to measure the impact of intervention (*ζ*) (figure 8*b*). The black curve depicts when there is no initiative (i.e. *ζ* = 0). The depreciation is more than the accumulation of income, and so income falls behind positive growth even at lower *r*_*H*_ ∼ 0.1. As the public health authority allocates more funds (say *ζ* = 15), the accumulation starts increasing which keeps the economic development consistently positive even at higher *r*_*H*_ (the extreme right yellow curve). Thus, a timely and right amount of public health initiatives may protect the population from the poverty trap and put the population back on the track of economic advancement.

## 4. Conclusion

The threat of antimicrobial resistance (AMR) is undoubtedly growing at an alarming rate and the situation is perhaps aggravated in developing countries due to gross misuse of antibiotics, mainly through self-medication (SM) [1,2]. Overuse of antibiotics in particular and self-medication in general are problems that involve social, behavioural and health issues, and economies of many LIMC and LICs [6,8,9]. A large percentage of the global population practice SM, especially antimicrobial, before seeking proper care at public service, which has been recognized as a major contributing factor to the current burden of antibiotic drug-resistance worldwide [63,64]. Van Boeckel *et al*. reported that India was the largest consumer of antibiotics with 12.9 × 10^9^ units (10.7 units per person) sold in the year 2010. Between 2000 and 2010, BRICS countries accounted for 76% of the overall increase in global antibiotic consumption, with India accounting for 23% [21,65].

Many empirical studies specify that lack of education and awareness in the population and lower socio-economic growth motivate self-medication. This is also reflected in the divergence of community-acquired resistance between developing and developed nations (figure 1) [63,66]. Using evolutionary game theory, we develop a framework in this analysis to understand the co-evolving dynamics of disease epidemiology and behavioural interactions that motivate individuals’ self-medication under several epidemiological and socio-economic scenarios. The game-theoretic perspective not only focuses our attention on the precise nature of adaptive human behaviour, which can guide our expectations about the emergence of antibiotic resistance, but also provides some insights to public health authorities about key factors in such an ever-increasing burden of resistance.

Although the nonlinear relationship between health and income is mentioned earlier in both empirical and theoretical studies [50,53], the present study emphasizes feedback dynamics between the economy and health, generating a self-reinforcing cycle mediated by individuals’ decision-making in self-medication. Our model explores dynamical regimes such as diagnostic rate, treatment effectiveness, risk perceptions and awareness to identify when and why individuals choose to self-medicate. Furthermore, we emphasize that increased and timely public health initiatives, such as providing financial risk protection through universal health coverage or insurance mechanisms to reduce treatment costs and diagnostics, can break this self-reinforcing cycle, recovering the population from economic downfall and continuous morbidity caused by antibiotic drug-resistance [6]. Various international funding agencies such as GHIT, MOFA AND MHLW in Japan, and the Bill & Melinda Gates foundations in the USA, are working to combat drug-resistance in developing countries by providing large funds to the developing nations every year [67–69]. In contrast to our earlier research paper [6], this paper explains the *pathways* of impact in real-world scenarios. This modelling work predicts that financial risk protection effectively reduces the cost of treatment thereby providing an opportunity to reduce self-medication, which consequently improves compliance with a full course of antibiotics, reducing the probability of the emergence of drug-resistance. This dynamic interrelationship explains how it potentially can reduce the magnitude of the problem of drug-resistance worldwide.

Every mathematical model is based upon simpler assumptions, and our self-medication game model is no exception. An important social dilemma impacting the burden of drug-resistance is the prescribing behaviour of community physicians. Chen & Fu [40], and Colman *et al.* [41] developed models to illustrate the irrational prescribing decisions by doctors that increase the level of antibiotic resistance in the community, and concluded that the burden may therefore be inevitable unless some means are found of modifying the payoffs of this potentially catastrophic social dilemma. However, a more complex model considering the physician-patients-population triad might require us to explore the social dilemma at both an individual and physician level and find out the consequences of the burden of drug-resistance. Also, considering the nonlinear production form in the constant-elasticity substitution (CES) function is more realistic as economic production depends on many factors other than labour and capital. Additionally, individuals’ risk perception depends on several social norms, media exposure, etc.—inclusion of which might expand the practicality and feasibility of the model. Analysis of the model can be studied by considering other socio-economic parameters like hygiene level, living conditions and nutrition.

Nonetheless, our work is the first that provides a framework to describe self-medication as a game, exploring individuals’ strategic decision-making in health practice and its externalities on society. Individuals always maximize their payoff while ignoring population-level externalities when making such decisions [70,71]. That is why it is critical to understand the interactions between resistance prevalence, treatment cost, and individual perceptions of self-medication, as this may aid in the management of antibiotic utilization for the benefit of individual health in the community.

## Data accessibility

All scripts used in this study are openly accessible through https://github.com/StochasticBiology/boolean-efflux.git. The data are provided in electronic supplementary material [72].
