# Supplementary material for: Disparity in socio-economic status explains the pattern of self-medication of antibiotics in India: understanding from game-theoretic perspective
Source: R Soc Open Sci. 2022 Feb 9;9(2):211872. doi: 10.1098/rsos.211872 (PMC8826305; doi:10.1098/rsos.211872)
Supplement: Supplementary file 1 [file rsos211872_review_history.pdf]

THE ROYAL SOCIETY
PUBLISHING

ROYAL SOCIETY
OPEN SCIENCE

Disparity in socio-economic status explains the pattern of self-medication of antibiotics in India: understanding from game-theoretic perspective

Bhawna Malik, Habib Hasan Farooqui and Samit Bhattacharyya

Article citation details
R. Soc. open sci. **9**: 211872.
http://dx.doi.org/10.1098/rsos.211872

Review timeline

Original submission:	12 August 2021
1st revised submission:	6 September 2021
2nd revised submission:	30 November 2021
3rd revised submission:	30 December 2021
Final acceptance:	11 January 2022

Note: Reports are unedited and appear as submitted by the referee. The review history appears in chronological order.

Note: This manuscript was transferred from another Royal Society journal without peer review.

Review History

Decision letter (RSOS-211262.R0)

We hope you are keeping well at this difficult and unusual time. We continue to value your support of the journal in these challenging circumstances. If Royal Society Open Science can assist you at all, please don't hesitate to let us know at the email address below.

Dear Dr Bhattacharyya

The Editors assigned to your paper RSOS-211262 "Disparity in socioeconomic status explains the pattern of self-medication of antibiotics in India: understanding from game-theoretic perspective" have made a decision based on their reading of the paper and any comments received from reviewers.

Regrettably, in view of the reports received, the manuscript has been rejected in its current form. However, a new manuscript may be submitted which takes into consideration these comments.

Reports © 2022 The Reviewers; Decision Letters © 2022 The Reviewers and Editors; Responses © 2022 The Reviewers, Editors and Authors. Published by the Royal Society under the terms of the Creative Commons Attribution License http://creativecommons.org/licenses/by/4.0/, which permits unrestricted use, provided the original author and source are credited

We invite you to respond to the comments supplied below and prepare a resubmission of your manuscript. Below the referees' and Editors' comments (where applicable) we provide additional requirements. We provide guidance below to help you prepare your revision.

Please note that resubmitting your manuscript does not guarantee eventual acceptance, and we do not generally allow multiple rounds of revision and resubmission, so we urge you to make every effort to fully address all of the comments at this stage. If deemed necessary by the Editors, your manuscript will be sent back to one or more of the original reviewers for assessment. If the original reviewers are not available, we may invite new reviewers.

Please resubmit your revised manuscript and required files (see below) no later than 02-Mar-2022. Note: the ScholarOne system will 'lock' if resubmission is attempted on or after this deadline. If you do not think you will be able to meet this deadline, please contact the editorial office immediately.

Please note article processing charges apply to papers accepted for publication in Royal Society Open Science (https://royalsocietypublishing.org/rsos/charges). Charges will also apply to papers transferred to the journal from other Royal Society Publishing journals, as well as papers submitted as part of our collaboration with the Royal Society of Chemistry (https://royalsocietypublishing.org/rsos/chemistry). Fee waivers are available but must be requested when you submit your manuscript (https://royalsocietypublishing.org/rsos/waivers).

Thank you for submitting your manuscript to Royal Society Open Science and we look forward to receiving your resubmission. If you have any questions at all, please do not hesitate to get in touch.

Kind regards,
Royal Society Open Science Editorial Office
Royal Society Open Science
openscience@royalsociety.org

Associate Editor Comments to Author:
Associate Editor
Comments to the Author:
The article is mostly well written and describes an important topic. My main concern is the article's similarity to a previously published paper: Malik B, Bhattacharyya S. 2019 Antibiotic drug-resistance as a complex system driven by socio-economic growth and antibiotic misuse. Scientific reports 9, 1–12. Although the model is slightly different in the paper we are reviewing, the underlying ideas seem very similar and it would have been useful to see a more thorough discussion of the additional contributions that this new paper makes to the literature.

===PREPARING YOUR MANUSCRIPT===

Your revised paper should include the changes requested by the referees and Editors of your manuscript. You should provide two versions of this manuscript and both versions must be provided in an editable format:
one version identifying all the changes that have been made (for instance, in coloured highlight, in bold text, or tracked changes);
a 'clean' version of the new manuscript that incorporates the changes made, but does not highlight them. This version will be used for typesetting if your manuscript is accepted.

Please ensure that any equations included in the paper are editable text and not embedded images.

Please ensure that you include an acknowledgements' section before your reference list/bibliography. This should acknowledge anyone who assisted with your work, but does not qualify as an author per the guidelines at https://royalsociety.org/journals/ethics-policies/openness/.

While not essential, it will speed up the preparation of your manuscript proof if accepted if you format your references/bibliography in Vancouver style (please see https://royalsociety.org/journals/authors/author-guidelines/#formatting). You should include DOIs for as many of the references as possible.

If you have been asked to revise the written English in your submission as a condition of publication, you must do so, and you are expected to provide evidence that you have received language editing support. The journal would prefer that you use a professional language editing service and provide a certificate of editing, but a signed letter from a colleague who is a native speaker of English is acceptable. Note the journal has arranged a number of discounts for authors using professional language editing services (https://royalsociety.org/journals/authors/benefits/language-editing/).

===PREPARING YOUR REVISION IN SCHOLARONE===

To revise your manuscript, log into https://mc.manuscriptcentral.com/rsos and enter your Author Centre - this may be accessed by clicking on "Author" in the dark toolbar at the top of the page (just below the journal name). You will find your manuscript listed under "Manuscripts with Decisions". Under "Actions", click on "Create a Revision".

Attach your point-by-point response to referees and Editors at Step 1 'View and respond to decision letter'. This document should be uploaded in an editable file type (.doc or .docx are preferred). This is essential.

Please ensure that you include a summary of your paper at Step 2 'Type, Title, & Abstract'. This should be no more than 100 words to explain to a non-scientific audience the key findings of your research. This will be included in a weekly highlights email circulated by the Royal Society press office to national UK, international, and scientific news outlets to promote your work.

At Step 3 'File upload' you should include the following files:
-- Your revised manuscript in editable file format (.doc, .docx, or .tex preferred). You should upload two versions:
1) One version identifying all the changes that have been made (for instance, in coloured highlight, in bold text, or tracked changes);
2) A 'clean' version of the new manuscript that incorporates the changes made, but does not highlight them.
-- An individual file of each figure (EPS or print-quality PDF preferred [either format should be produced directly from original creation package], or original software format).
-- An editable file of each table (.doc, .docx, .xls, .xlsx, or .csv).
-- An editable file of all figure and table captions.
Note: you may upload the figure, table, and caption files in a single Zip folder.
-- Any electronic supplementary material (ESM).
-- If you are requesting a discretionary waiver for the article processing charge, the waiver form must be included at this step.

-- If you are providing image files for potential cover images, please upload these at this step, and inform the editorial office you have done so. You must hold the copyright to any image provided.
-- A copy of your point-by-point response to referees and Editors. This will expedite the preparation of your proof.

At Step 6 'Details & comments', you should review and respond to the queries on the electronic submission form. In particular, we would ask that you do the following:
-- Ensure that your data access statement meets the requirements at https://royalsociety.org/journals/authors/author-guidelines/#data. You should ensure that you cite the dataset in your reference list. If you have deposited data etc in the Dryad repository, please include both the 'For publication' link and 'For review' link at this stage.
-- If you are requesting an article processing charge waiver, you must select the relevant waiver option (if requesting a discretionary waiver, the form should have been uploaded at Step 3 'File upload' above).
-- If you have uploaded ESM files, please ensure you follow the guidance at https://royalsociety.org/journals/authors/author-guidelines/#supplementary-material to include a suitable title and informative caption. An example of appropriate titling and captioning may be found at https://figshare.com/articles/Table_S2_from_Is_there_a_trade-off_between_peak_performance_and_performance_breadth_across_temperatures_for_aerobic_scope_in_teleost_fishes_/3843624.

At Step 7 'Review & submit', you must view the PDF proof of the manuscript before you will be able to submit the revision. Note: if any parts of the electronic submission form have not been completed, these will be noted by red message boxes.

Author's Response to Decision Letter for (RSOS-211262.R0)

See Appendix A.

RSOS-211443.R0

Review form: Reviewer 1

Is the manuscript scientifically sound in its present form?
No

Are the interpretations and conclusions justified by the results?
No

Is the language acceptable?
No

Do you have any ethical concerns with this paper?
No

Have you any concerns about statistical analyses in this paper?
Yes

Recommendation?
Major revision is needed (please make suggestions in comments)

Comments to the Author(s)
See attached file (Appendix B).

Review form: Reviewer 2

Is the manuscript scientifically sound in its present form?
Yes

Are the interpretations and conclusions justified by the results?
Yes

Is the language acceptable?
Yes

Do you have any ethical concerns with this paper?
No

Have you any concerns about statistical analyses in this paper?
No

Recommendation?
Accept with minor revision (please list in comments)

Comments to the Author(s)
- valuable contribution. Would have been interesting to contrast the patient perspective (self-medication) to the physician's perspective (prescribing antibiotics) in "Colman AM, Krockow EM, Chattoe-Brown E, Tarrant C (2019) Medical prescribing and antibiotic resistance: A game-theoretic analysis of a potentially catastrophic social dilemma. PLoS ONE 14(4): e0215480. https://doi.org/10.1371/journal.pone.0215480" or "Chen X, Fu F. 2018 Social learning of prescribing behavior can promote population optimum
of antibiotic use. Frontiers in Physics 6, 139" since game theory is also used to model the physicians' decisions. This may better highlight the authors' contributions
- recommend making the conclusion more concise and clear

Decision letter (RSOS-211443.R0)

We hope you are keeping well at this difficult and unusual time. We continue to value your support of the journal in these challenging circumstances. If Royal Society Open Science can assist you at all, please don't hesitate to let us know at the email address below.

Dear Dr Bhattacharyya

The Editors assigned to your paper RSOS-211443 "Disparity in socioeconomic status explains the pattern of self-medication of antibiotics in India: understanding from game-theoretic perspective"

have made a decision based on their reading of the paper and any comments received from reviewers.

Regrettably, in view of the reports received, the manuscript has been rejected in its current form. However, a new manuscript may be submitted which takes into consideration these comments.

We invite you to respond to the comments supplied below and prepare a resubmission of your manuscript. Below the referees' and Editors' comments (where applicable) we provide additional requirements. We provide guidance below to help you prepare your revision.

Please note that resubmitting your manuscript does not guarantee eventual acceptance, and we do not generally allow multiple rounds of revision and resubmission, so we urge you to make every effort to fully address all of the comments at this stage. If deemed necessary by the Editors, your manuscript will be sent back to one or more of the original reviewers for assessment. If the original reviewers are not available, we may invite new reviewers.

Please resubmit your revised manuscript and required files (see below) no later than 21-Apr-2022. Note: the ScholarOne system will 'lock' if resubmission is attempted on or after this deadline. If you do not think you will be able to meet this deadline, please contact the editorial office immediately.

Please note article processing charges apply to papers accepted for publication in Royal Society Open Science (https://royalsocietypublishing.org/rsos/charges). Charges will also apply to papers transferred to the journal from other Royal Society Publishing journals, as well as papers submitted as part of our collaboration with the Royal Society of Chemistry (https://royalsocietypublishing.org/rsos/chemistry). Fee waivers are available but must be requested when you submit your manuscript (https://royalsocietypublishing.org/rsos/waivers).

Thank you for submitting your manuscript to Royal Society Open Science and we look forward to receiving your resubmission. If you have any questions at all, please do not hesitate to get in touch.

Kind regards,
Royal Society Open Science Editorial Office
Royal Society Open Science
openscience@royalsociety.org

on behalf of Professor Christine Currie (Associate Editor) and Pietro Cicuta (Subject Editor)
openscience@royalsociety.org

Associate Editor Comments to Author (Professor Christine Currie):
Comments to the Author:
The referees have raised some major concerns about your article and the first referee has suggested some significant changes that are needed before publication. In addition, the quality of the writing still needs improving. As a result, I feel that there is a considerable amount of work needed on this article before RSOS could accept it.

Reviewer comments to Author:
Reviewer: 1
Comments to the Author(s)
See attached file

Reviewer: 2
Comments to the Author(s)
- valuable contribution. Would have been interesting to contrast the patient perspective (self-medication) to the physician's perspective (prescribing antibiotics) in "Colman AM, Krockow EM, Chattoe-Brown E, Tarrant C (2019) Medical prescribing and antibiotic resistance: A game-theoretic analysis of a potentially catastrophic social dilemma. PLoS ONE 14(4): e0215480. https://doi.org/10.1371/journal.pone.0215480" or "Chen X, Fu F. 2018 Social learning of prescribing behavior can promote population optimum
of antibiotic use. Frontiers in Physics 6, 139" since game theory is also used to model the physicians' decisions. This may better highlight the authors' contributions
- recommend making the conclusion more concise and clear

===PREPARING YOUR MANUSCRIPT===

Your revised paper should include the changes requested by the referees and Editors of your manuscript. You should provide two versions of this manuscript and both versions must be provided in an editable format:
one version identifying all the changes that have been made (for instance, in coloured highlight, in bold text, or tracked changes);
a 'clean' version of the new manuscript that incorporates the changes made, but does not highlight them. This version will be used for typesetting if your manuscript is accepted.

Please ensure that any equations included in the paper are editable text and not embedded images.

Please ensure that you include an acknowledgements' section before your reference list/bibliography. This should acknowledge anyone who assisted with your work, but does not qualify as an author per the guidelines at https://royalsociety.org/journals/ethics-policies/openness/.

While not essential, it will speed up the preparation of your manuscript proof if accepted if you format your references/bibliography in Vancouver style (please see https://royalsociety.org/journals/authors/author-guidelines/#formatting). You should include DOIs for as many of the references as possible.

If you have been asked to revise the written English in your submission as a condition of publication, you must do so, and you are expected to provide evidence that you have received language editing support. The journal would prefer that you use a professional language editing service and provide a certificate of editing, but a signed letter from a colleague who is a native speaker of English is acceptable. Note the journal has arranged a number of discounts for authors using professional language editing services (https://royalsociety.org/journals/authors/benefits/language-editing/).

===PREPARING YOUR REVISION IN SCHOLARONE===

To revise your manuscript, log into https://mc.manuscriptcentral.com/rsos and enter your Author Centre - this may be accessed by clicking on "Author" in the dark toolbar at the top of the page (just below the journal name). You will find your manuscript listed under "Manuscripts with Decisions". Under "Actions", click on "Create a Revision".

Attach your point-by-point response to referees and Editors at Step 1 'View and respond to decision letter'. This document should be uploaded in an editable file type (.doc or .docx are preferred). This is essential.

Please ensure that you include a summary of your paper at Step 2 'Type, Title, & Abstract'. This should be no more than 100 words to explain to a non-scientific audience the key findings of your research. This will be included in a weekly highlights email circulated by the Royal Society press office to national UK, international, and scientific news outlets to promote your work.

At Step 3 'File upload' you should include the following files:
-- Your revised manuscript in editable file format (.doc, .docx, or .tex preferred). You should upload two versions:
1) One version identifying all the changes that have been made (for instance, in coloured highlight, in bold text, or tracked changes);
2) A 'clean' version of the new manuscript that incorporates the changes made, but does not highlight them.
-- An individual file of each figure (EPS or print-quality PDF preferred [either format should be produced directly from original creation package], or original software format).
-- An editable file of each table (.doc, .docx, .xls, .xlsx, or .csv).
-- An editable file of all figure and table captions.
Note: you may upload the figure, table, and caption files in a single Zip folder.
-- Any electronic supplementary material (ESM).
-- If you are requesting a discretionary waiver for the article processing charge, the waiver form must be included at this step.
-- If you are providing image files for potential cover images, please upload these at this step, and inform the editorial office you have done so. You must hold the copyright to any image provided.
-- A copy of your point-by-point response to referees and Editors. This will expedite the preparation of your proof.

At Step 6 'Details & comments', you should review and respond to the queries on the electronic submission form. In particular, we would ask that you do the following:
-- Ensure that your data access statement meets the requirements at https://royalsociety.org/journals/authors/author-guidelines/#data. You should ensure that you cite the dataset in your reference list. If you have deposited data etc in the Dryad repository, please include both the 'For publication' link and 'For review' link at this stage.
-- If you are requesting an article processing charge waiver, you must select the relevant waiver option (if requesting a discretionary waiver, the form should have been uploaded at Step 3 'File upload' above).
-- If you have uploaded ESM files, please ensure you follow the guidance at https://royalsociety.org/journals/authors/author-guidelines/#supplementary-material to include a suitable title and informative caption. An example of appropriate titling and captioning may be found at https://figshare.com/articles/Table_S2_from_Is_there_a_trade-off_between_peak_performance_and_performance_breadth_across_temperatures_for_aerobic_scope_in_teleost_fishes_/3843624.

At Step 7 'Review & submit', you must view the PDF proof of the manuscript before you will be able to submit the revision. Note: if any parts of the electronic submission form have not been completed, these will be noted by red message boxes.

Author's Response to Decision Letter for (RSOS-211443.R0)

See Appendix C.

RSOS-211872.R0

Review form: Reviewer 1

Is the manuscript scientifically sound in its present form?
Yes

Are the interpretations and conclusions justified by the results?
Yes

Is the language acceptable?
Yes

Do you have any ethical concerns with this paper?
No

Have you any concerns about statistical analyses in this paper?
No

Recommendation?
Accept as is

Comments to the Author(s)
The authors have satisfactorily answered all my queries. This work is now suitable for publication. Congratulations!

Review form: Reviewer 2

Is the manuscript scientifically sound in its present form?
Yes

Are the interpretations and conclusions justified by the results?
Yes

Is the language acceptable?
Yes

Do you have any ethical concerns with this paper?
No

Have you any concerns about statistical analyses in this paper?
No

Recommendation?
Accept with minor revision (please list in comments)

Comments to the Author(s)
1. Figure captions can me more informative and highlight key takeaway from figure (especially when there is comparison of sub-figures like in Fig 3)

2. Requires some more proofreading. Below are some minor improvements

Page 9 of 37 (10 of 40):
Line 31: "are developing countries show highest drug consumption from 2000 to 2010" -- 'are developing countries that showed their highest'.
Line 58: "incurs a huge cost on nations economy" -- either 'on a nation's economy' or 'on nations' economies'.

Page 10 of 37 (11 of 40)
Line 9: "As predicted, it would lose 1.1-3.8$ of its annual gross domestic product (GDP)" -- the dollar sign always precedes the figure.
Line 11: "will suffer more in-comparison to developed nations" -- 'in comparison' is not a compound adjective and thus does not need a hyphen.

Page 16 of 37 (17 of 40)
Line 51: "the budget is released proportional to the current prevalence of resistance, – meaning higher the prevalence, more active the public health is – in allocating funds" -- Parenthetical content should be enclosed between two em dashes (not en dashes) with no spaces and any surrounding punctuation must be omitted, lime so: 'the budget is released proportional to the current prevalence of resistance—meaning higher the prevalence, more active the public health is—in allocating funds'

Decision letter (RSOS-211872.R0)

We hope you are keeping well at this difficult and unusual time. We continue to value your support of the journal in these challenging circumstances. If Royal Society Open Science can assist you at all, please don't hesitate to let us know at the email address below.

Dear Dr Bhattacharyya

On behalf of the Editors, we are pleased to inform you that your Manuscript RSOS-211872 "Disparity in socioeconomic status explains the pattern of self-medication of antibiotics in India: understanding from game-theoretic perspective" has been accepted for publication in Royal Society Open Science subject to minor revision in accordance with the referees' reports. Please find the referees' comments along with any feedback from the Editors below my signature.

We invite you to respond to the comments and revise your manuscript. Below the referees' and Editors' comments (where applicable) we provide additional requirements. Final acceptance of your manuscript is dependent on these requirements being met. We provide guidance below to help you prepare your revision.

Please submit your revised manuscript and required files (see below) no later than Friday 14 January 2022. Note: the ScholarOne system will 'lock' if submission of the revision is attempted after the deadline. If you do not think you will be able to meet this deadline please contact the editorial office immediately.

Please note article processing charges apply to papers accepted for publication in Royal Society Open Science (https://royalsocietypublishing.org/rsos/charges). Charges will also apply to papers transferred to the journal from other Royal Society Publishing journals, as well as papers submitted as part of our collaboration with the Royal Society of Chemistry

(https://royalsocietypublishing.org/rsos/chemistry). Fee waivers are available but must be requested when you submit your revision (https://royalsocietypublishing.org/rsos/waivers).

Thank you for submitting your manuscript to Royal Society Open Science and we look forward to receiving your revision. If you have any questions at all, please do not hesitate to get in touch.

Kind regards,
Royal Society Open Science Editorial Office
Royal Society Open Science
openscience@royalsociety.org

on behalf of Professor Christine Currie (Associate Editor) and Pietro Cicuta (Subject Editor)
openscience@royalsociety.org

Associate Editor Comments to Author (Professor Christine Currie):
Associate Editor
Comments to the Author:
The paper is very close to completion but I agree with them that there is a need for a final read through to correct grammatical and typographical errors. Some of these will be picked up at the proofing stage but it is generally easier for authors to correct them before it gets to proofing.

Reviewer comments to Author:
Reviewer: 1
Comments to the Author(s)
The authors have satisfactorily answered all my queries. This work is now suitable for publication. Congratulations!

Reviewer: 2
Comments to the Author(s)
1. Figure captions can me more informative and highlight key takeaway from figure (especially when there is comparison of sub-figures like in Fig 3)

2. Requires some more proofreading. Below are some minor improvements

Page 9 of 37 (10 of 40):
Line 31: "are developing countries show highest drug consumption from 2000 to 2010" -- 'are developing countries that showed their highest'.
Line 58: "incurs a huge cost on nations economy" -- either 'on a nation's economy' or 'on nations' economies'.

Page 10 of 37 (11 of 40)
Line 9: "As predicted, it would lose 1.1-3.8$ of its annual gross domestic product (GDP)" -- the dollar sign always precedes the figure.
Line 11: "will suffer more in-comparison to developed nations" -- 'in comparison' is not a compound adjective and thus does not need a hyphen.

Page 16 of 37 (17 of 40)
Line 51: "the budget is released proportional to the current prevalence of resistance, – meaning higher the prevalence, more active the public health is – in allocating funds" -- Parenthetical content should be enclosed between two em dashes (not en dashes) with no spaces and any surrounding punctuation must be omitted, lime so: 'the budget is released proportional to the

current prevalence of resistance—meaning higher the prevalence, more active the public health is—in allocating funds'

===PREPARING YOUR MANUSCRIPT===

Your revised paper should include the changes requested by the referees and Editors of your manuscript.

You should provide two versions of this manuscript and both versions must be provided in an editable format:
one version should clearly identify all the changes that have been made (for instance, in coloured highlight, in bold text, or tracked changes);
a 'clean' version of the new manuscript that incorporates the changes made, but does not highlight them. This version will be used for typesetting.

Please ensure that any equations included in the paper are editable text and not embedded images.

Please ensure that you include an acknowledgements' section before your reference list/bibliography. This should acknowledge anyone who assisted with your work, but does not qualify as an author per the guidelines at https://royalsociety.org/journals/ethics-policies/openness/.

While not essential, it will speed up the preparation of your manuscript proof if you format your references/bibliography in Vancouver style (please see https://royalsociety.org/journals/authors/author-guidelines/#formatting). You should include DOIs for as many of the references as possible.

If you have been asked to revise the written English in your submission as a condition of publication, you must do so, and you are expected to provide evidence that you have received language editing support. The journal would prefer that you use a professional language editing service and provide a certificate of editing, but a signed letter from a colleague who is a proficient user of English is acceptable. Note the journal has arranged a number of discounts for authors using professional language editing services (https://royalsociety.org/journals/authors/benefits/language-editing/).

===PREPARING YOUR REVISION IN SCHOLARONE===

To revise your manuscript, log into https://mc.manuscriptcentral.com/rsos and enter your Author Centre - this may be accessed by clicking on "Author" in the dark toolbar at the top of the page (just below the journal name). You will find your manuscript listed under "Manuscripts with Decisions". Under "Actions", click on "Create a Revision".

Attach your point-by-point response to referees and Editors at the 'View and respond to decision letter' step. This document should be uploaded in an editable file type (.doc or .docx are preferred). This is essential, and your manuscript will be returned to you if you do not provide it.

Please ensure that you include a summary of your paper at the 'Type, Title, & Abstract' step. This should be no more than 100 words to explain to a non-scientific audience the key findings of your research. This will be included in a weekly highlights email circulated by the Royal Society press office to national UK, international, and scientific news outlets to promote your work. An effective summary can substantially increase the readership of your paper.

At the 'File upload' step you should include the following files:
-- Your revised manuscript in editable file format (.doc, .docx, or .tex preferred). You should upload two versions:
1) One version identifying all the changes that have been made (for instance, in coloured highlight, in bold text, or tracked changes);
2) A 'clean' version of the new manuscript that incorporates the changes made, but does not highlight them.
-- An individual file of each figure (EPS or print-quality PDF preferred [either format should be produced directly from original creation package], or original software format).
-- An editable file of each table (.doc, .docx, .xls, .xlsx, or .csv).
-- An editable file of all figure and table captions.
Note: you may upload the figure, table, and caption files in a single Zip folder.
-- Any electronic supplementary material (ESM).
-- If you are requesting a discretionary waiver for the article processing charge, the waiver form must be included at this step.
-- If you are providing image files for potential cover images, please upload these at this step, and inform the editorial office you have done so. You must hold the copyright to any image provided.
-- A copy of your point-by-point response to referees and Editors. This will expedite the preparation of your proof.

At the 'Details & comments' step, you should review and respond to the queries on the electronic submission form. In particular, we would ask that you do the following:
-- Ensure that your data access statement meets the requirements at https://royalsociety.org/journals/authors/author-guidelines/#data. You should ensure that you cite the dataset in your reference list. If you have deposited data etc in the Dryad repository, please only include the 'For publication' link at this stage. You should remove the 'For review' link.
-- If you are requesting an article processing charge waiver, you must select the relevant waiver option (if requesting a discretionary waiver, the form should have been uploaded, see 'File upload' above).
-- If you have uploaded any electronic supplementary (ESM) files, please ensure you follow the guidance at https://royalsociety.org/journals/authors/author-guidelines/#supplementary-material to include a suitable title and informative caption. An example of appropriate titling and captioning may be found at https://figshare.com/articles/Table_S2_from_Is_there_a_trade-off_between_peak_performance_and_performance_breadth_across_temperatures_for_aerobic_scope_in_teleost_fishes_/3843624.

At the 'Review & submit' step, you must view the PDF proof of the manuscript before you will be able to submit the revision. Note: if any parts of the electronic submission form have not been completed, these will be noted by red message boxes - you will need to resolve these errors before you can submit the revision.

Author's Response to Decision Letter for (RSOS-211872.R0)

See Appendix D.

Decision letter (RSOS-211872.R1)

We hope you are keeping well at this difficult and unusual time. We continue to value your support of the journal in these challenging circumstances. If Royal Society Open Science can assist you at all, please don't hesitate to let us know at the email address below.

Dear Dr Bhattacharyya,

It is a pleasure to accept your manuscript entitled "Disparity in socioeconomic status explains the pattern of self-medication of antibiotics in India: understanding from game-theoretic perspective" in its current form for publication in Royal Society Open Science. The comments of the reviewer(s) who reviewed your manuscript are included at the foot of this letter.

If you have not already done so, please ensure that you send to the editorial office an editable version of your accepted manuscript, and individual files for each figure and table included in your manuscript. You can send these in a zip folder if more convenient. Failure to provide these files may delay the processing of your proof.

Please remember to make any data sets or code libraries 'live' prior to publication, and update any links as needed when you receive a proof to check - for instance, from a private 'for review' URL to a publicly accessible 'for publication' URL. It is good practice to also add data sets, code and other digital materials to your reference list.

Our payments team will be in touch shortly if you are required to pay a fee for the publication of the paper (if you have any queries regarding fees, please see https://royalsocietypublishing.org/rsos/charges or contact authorfees@royalsociety.org).

The proof of your paper will be available for review using the Royal Society online proofing system and you will receive details of how to access this in the near future from our production office (openscience_proofs@royalsociety.org). We aim to maintain rapid times to publication after acceptance of your manuscript and we would ask you to please contact both the production office and editorial office if you are likely to be away from e-mail contact to minimise delays to publication. If you are going to be away, please nominate a co-author (if available) to manage the proofing process, and ensure they are copied into your email to the journal.

Please see the Royal Society Publishing guidance on how you may share your accepted author manuscript at https://royalsociety.org/journals/ethics-policies/media-embargo/. After publication, some additional ways to effectively promote your article can also be found here https://royalsociety.org/blog/2020/07/promoting-your-latest-paper-and-tracking-your-results/.

Thank you for your fine contribution. On behalf of the Editors of Royal Society Open Science, we look forward to your continued contributions to the Journal.

Kind regards,
Royal Society Open Science Editorial Office
Royal Society Open Science
openscience@royalsociety.org

on behalf of Professor Christine Currie (Associate Editor) and Pietro Cicuta (Subject Editor)
openscience@royalsociety.org

Associate Editor Comments to Author (Professor Christine Currie):
Associate Editor
Comments to the Author:
Congratulations! This is a very interesting paper that I am sure will be successful within the journal. Many thanks for making these last few corrections. I'm happy to suggest that the paper be accepted.

Your revision has raised one very minor query in the last paragraph of the conclusion. Here you refer to papers about vaccination and then draw a conclusion about self-medication. There seemed a slight disconnect which you may want to consider when you are at the proofing stage. I suspect it would only involve changing a few words if you did decide to amend it.

Follow Royal Society Publishing on Twitter: @RSocPublishing
Follow Royal Society Publishing on Facebook:
https://www.facebook.com/RoyalSocietyPublishing.FanPage/
Read Royal Society Publishing's blog:
https://royalsociety.org/blog/blogsearchpage/?category=Publishing

Appendix A

Reply to review comments (ID RSOS-211262)

Associate Editor Comments to Author:

The article is mostly well written and describes an important topic. My main concern is the article's similarity to a previously published paper: Malik B, Bhattacharyya S. 2019 Antibiotic drug-resistance as a complex system driven by socio-economic growth and antibiotic misuse. Scientific reports 9, 1–12. Although the model is slightly different in the paper we are reviewing, the underlying ideas seem very similar and it would have been useful to see a more thorough discussion of the additional contributions that this new paper makes to the literature.

Many thanks to the Editor for the comments and suggestions. In the earlier paper [6], although we used same framework of integrated system of infectious disease, socioeconomic growth, and antibiotic misuse to explore the dynamics of emergence of drug resistance, we did not consider human behaviour in *self-medication* and how economy and disease prevalence motivate such behaviour. Earlier model was simple, and No game theoretic aspect was considered.

Here, in current manuscript, we have explored the human behaviour in self-medication by developing game theoretic model. We have investigated the emergence of resistance under the mechanistic frame of human behaviour (i.e., *self-medication*) that mediates the antibiotic misuse. This gives us opportunity to understand the relationship between population level medication behaviour and its consequence on emergence on drug resistance. The current model is more complex than previous model.

We have added paragraphs in *Introduction* and in *Conclusion* separately to point out clearly the potentiality of the present work compare to earlier and additional contributions from it.

Appendix B

In the study by Bhawna et al. the authors develop and explore a mathematical model describing AMR spread while considering the practice of self-medicating Vs. hospital treatments of antibiotics. The model includes a compartmental SIR-like component, an economic growth component, and an evolutionary game theory component describing behavioural changes. This is an interesting paper, creating an intricate, multi-component model with relevant results. It should be interesting to the readers of JRSOS. However, I have some major comments that should be addressed if it is to be published.

Major comments:

- The text needs proofreading and grammar corrections. I commented some examples below, but many more changes are needed.
- Figure 1 – Is it a statistically significant trend? Some indication of variance (e.g. CIs of the coefficients) should be presented.
- Methods: "For example, individuals infected with sensitive strain report mild symptoms, whereas severe symptoms are developed upon infection by a resistant strain." – is this assumption reasonable? Antibiotic sensitive strains are definitely not less virulent than resistant strains. They are easier to treat but this is a different assumption. Please justify.
- Equation 2.1: the term mz appears in the equation yet z is only explained much later; m is defined as the "susceptibility to the resistant strain", yet this is unclear – in which units? How do you explain it physiologically, is that related to the host immunity?
- Supplementary: you assume that $1/\eta 1<1/\gamma 1< 1/\eta 2$. With respect to η, it is clear. However, why do you assume that the average duration of colonization of the sensitive strain ($1/\gamma 1$) shorter than the recovery period of an individual treated at home ($1/\eta 2$)?
- You present equations of the economic model with no explanation of parameters in the main text. It is extremely hard to follow.
- You assume that economic growth is impeded by infections and you support this assumption by various correlations. However, why can't this be a result of reverse causation? I.e. that low economic status of a country leads to more infections due to a multitude of medical and behavioural reasons.
- Figure 4: it would be useful to see the bacterial dynamics to better understand the dynamics of the self-medication frequency

- c_z is not defined, or I missed it. I think a parameter table in the main text will be useful (the on in the SI doesn't have c_z either).
- The Results have a lot of discussions woven into it. Perhaps rephrase as Results and Discussion if allowed by the journal. If not, then some of the text should be in a designated Discussion section.

<u>Minor comments:</u>

- Abstract: "and self-medicate more likely", self-medication is more likely
- Introduction: "It is also indicated that 80% of all antibiotics are purchased without any prescription in developing countries which is substantiated by the studies that the prevalence of self-medication in such developing nations is in the range of 12.7% to 95%" – phrasing
- Introduction: "It was estimated 16.8% of antimicrobials out of the total medicine sales worth $12.6 billion in India between 2013 and 2014 [21]." – unclear
- Figure 1 – it is not a correlation per se. It is an association measure from a regression formula.
- There have been several papers (not only this groups' regarding antibiotic usage and game theory in recent years. I think they should be briefly discussed and contrasted with the current analysis, to better understand the advances the authors make in this paper.
- Methods: please provide a list of the model parameters and their values in the main text, it is important enough information to appear there rather than the SI.
- Methods: "the elasticity parameters, –actually functions of strain" ?
- Results: "However, the socially optimum values decreases"
- Results: "These simulations indicate that public health can work towards inform individuals of realistic values of those parameters to improve the control of drug resistance." – Unclear how this should/can be done. Moreover, this belongs to the Discussion rather than the Results.
- Results: " there is exactly no difference"
- Results: "…the situation is much improved if the amount of financial investment is higher and provided in a timely, especially, at the…" – timely what?

Appendix C

Reply to reviewers' comments

(Manuscript ID: RSOS-211443)

November 2021

Associate Editor Comments to Author (Professor Christine Currie):

The referees have raised some major concerns about your article and the first referee has suggested some significant changes that are needed before publication. In addition, the quality of the writing still needs improving. As a result, I feel that there is a considerable amount of work needed on this article before RSOS could accept it.

– We thank the Editor for giving us opportunity to consider reviewers' suggestions and revise the paper. We have carefully considered each suggestion and improved the manuscript for more clarity, and presentation. Below, we reply them point-by-point:

Reviewer 1 comments

Major comments

- The text needs proofreading and grammar corrections. I commented some examples below, but many more changes are needed.

  – We thank reviewer for mentioning this. We revised the texts and corrected all typos and grammatical error.

- Figure 1 – Is it a statistically significant trend? Some indication of variance (e.g. CIs of the coefficients) should be presented.

  –Yes, we have now presented 95% confidence interval with the prediction trend in the data.

- Methods: "For example, individuals infected with sensitive strain report mild symptoms, whereas severe symptoms are developed upon infection by a resistant strain." – is this assumption reasonable? Antibiotic sensitive

strains are definitely not less virulent than resistant strains. They are easier to treat but this is a different assumption. Please justify.

We are not talking about virulence; we are saying that under natural progression of disease, the symptoms usually worsens over time when the pathogen is antibiotic resistant, and the patient may require another class or higher dose of antibiotic for cure. For example, the natural course of infection because of commensal pathogens such as E. Coli – a bacteria that usually live harmlessly in the gut of healthy people but can cause problems if they get into the bladder or other parts of the urinary tract – starts as an uncomplicated urinary tract infection (mild disease or symptoms) but can turn into severe infection (if the E. Coli bacteria becomes resistant to certain antibiotics, and continue to multiply and spread) which is much more difficult to treat."

- Equation 2.1: the term mz appears in the equation yet z is only explained much later; m is defined as the "susceptibility to the resistant strain", yet this is unclear – in which units?

  – We have now redefined the terms and explained it. It is the parameter that quantifies the sensitivity of the adopting hospital treatment to change in prevalence. $m \in (0, 1)$, and so unitless.

- Supplementary: you assume that $1/\eta_1 < 1/\gamma_1 < 1/\eta_2$. With respect to η, it is clear. However, why do you assume that the average duration of colonization of the sensitive strain ($1/\gamma_1$) shorter than the recovery period of an individual treated at home ($1/\eta_2$)?

  –We assumed that treated individuals recover faster (η_1) than at-home (i.e., natural) recovery (γ_1). However, if individuals use self-medication, it naturally delays the recovery (denoted by η_2) from infection.

- You present equations of the economic model with no explanation of parameters in the main text. It is extremely hard to follow.

  – We thank reviewer for raising this issue. Now we have added explanations to the terms used in economic model.

- You assume that economic growth is impeded by infections and you support this assumption by various correlations. However, why can't this be a result of reverse causation? I.e. that low economic status of a country leads to more infections due to a multitude of medical and behavioural reasons.

– This is true, and it is very much reflected in our model system. More infections are in fact, the consequence of the low economic conditions, and this happens by the irrational self-medications of antimicrobial in populations.

- Figure 4: it would be useful to see the bacterial dynamics to better understand the dynamics of the self-medication frequency.

  —We have changed the figure and added time series that exhibit the impact on resistant strain of population.

- c_z is not defined, or I missed it. I think a parameter table in the main text will be useful (the on in the SI doesn't have c_z either).

  — We have now placed the parameter table in the main file and defined the parameter c_z.

- The Results have a lot of discussions woven into it. Perhaps rephrase as Results and Discussion if allowed by the journal. If not, then some of the text should be in a designated Discussion section.

  — We have rephrased it as Results and Discussions, as common approach of article format of RSOS. Also, we have shorten the explanations of results and its implication in many places in the **Result and discussion** section.

Minor comments

- Abstract: "and self-medicate more likely", self-medication is more likely

  — Rephrased the line.

- Introduction: "It is also indicated that 80% of all antibiotics are purchased without any prescription in developing countries which is substantiated by the studies that the prevalence of self-medication in such developing nations is in the range of 12.7% to 95%" – phrasing

  — Rephrased the line.

- Introduction: "It was estimated 16.8% of antimicrobials out of the total medicine sales worth \$ 12.6 billion in India between 2013 and 2014 [21]." – unclear

  — Rewritten the sentence properly.

- Figure 1 – it is not a correlation per se. It is an association measure from a regression formula.

  — Changed the ”correlation” to correspondence.

- There have been several papers (not only this groups’ regarding antibiotic usage and game theory in recent years. I think they should be briefly discussed and contrasted with the current analysis, to better understand the advances the authors make in this paper.

  — Added recent papers and their discussions in Introduction and also in Conclusions sections.

- Methods: please provide a list of the model parameters and their values in the main text, it is important enough information to appear there rather than the SI.

  — We have placed parameter table in the main file.

- Methods: “the elasticity parameters, –actually functions of strain” ?

  We have rephrased this.

- Results: “However, the socially optimum values decreases”

  We have rephrased this.

- Results: “These simulations indicate that public health can work towards inform individuals of realistic values of those parameters to improve the control of drug resistance.” – Unclear how this should/can be done. Moreover, this belongs to the Discussion rather than the Results.

  We have rephrased this sentences. Also now the section *Results* has been rephrased *Results and discussion.*

- Results: ”there is exactly no difference”

  We have rephrased this.

- Results: “...the situation is much improved if the amount of financial investment is higher and provided in a timely, especially, at the...” – timely what?

  We have rephrased this.

Reviewer 2 comments

- valuable contribution. Would have been interesting to contrast the patient perspective (self-medication) to the physician's perspective (prescribing antibiotics) in "Colman AM, Krockow EM, Chattoe-Brown E, Tarrant C (2019) Medical prescribing and antibiotic resistance: A game-theoretic analysis of a potentially catastrophic social dilemma. PLoS ONE 14(4): e0215480. https://doi.org/10.1371/journal.pone.0215480" or "Chen X, Fu F. 2018 Social learning of prescribing behavior can promote population optimum of antibiotic use. Frontiers in Physics 6, 139" since game theory is also used to model the physicians' decisions. This may better highlight the authors' contributions.

  Thanks reviewer for raising this. We have incorporated this part in the discussion.

- recommend making the conclusion more concise and clear.

  We have now revised the conclusion and shortened!

Appendix D

Reply to reviewers' comments

(Manuscript ID: RSOS-211872)

December 2021

Associate Editor Comments to Author:

The paper is very close to completion but I agree with them that there is a need for a final read through to correct grammatical and typographical errors. Some of these will be picked up at the proofing stage but it is generally easier for authors to correct them before it gets to proofing.

Authors' response:—Thanks for the comments. The manuscript is now revised and read thoroughly to give better understanding. We have responded to all comments as given below.

Reviewer comments to Author:

Reviewer: 1

The authors have satisfactorily answered all my queries. This work is now suitable for publication. Congratulations!

Authors' response:—We would like to thank reviewer 1 for kind recommendation.

Reviewer: 2

1. Figure captions can me more informative and highlight key takeaway from figure (especially when there is comparison of sub-figures like in Fig 3).

Authors' response:—Thank you for the comment. In the revised manuscript, we have modified captions of all figures by incorporating key messages of explanation.

2. Requires some more proofreading. Below are some minor improvements

Page 9 of 37 (10 of 40): Line 31: “are developing countries show highest drug consumption from 2000 to 2010” – ’are developing countries that showed their highest’.

Line 58: “incurs a huge cost on nations economy” – either ’on a nation’s economy’ or ’on nations’ economies’.

Page 10 of 37 (11 of 40) Line 9: “As predicted, it would lose 1.1-3.8$ of its annual gross domestic product (GDP)” – the dollar sign always precedes the figure.

Line 11: “will suffer more in-comparison to developed nations” – ’in comparison’ is not a compound adjective and thus does not need a hyphen.

Page 16 of 37 (17 of 40) Line 51: “the budget is released proportional to the current prevalence of resistance, – meaning higher the prevalence, more active the public health is – in allocating funds” – Parenthetical content should be enclosed between two em dashes (not en dashes) with no spaces and any surrounding punctuation must be omitted, lime so: ’the budget is released proportional to the current prevalence of resistance—meaning higher the prevalence, more active the public health is—in allocating funds’.

Authors’ response:—We have considered all above issues mentioned by the reviewer 2, and also other typographical and grammatical errors in the paper highlighted in boldfaces.
